# Anomalous aortic origin of the coronary arteries in a 12-year-old male: a case report

**DOI:** 10.1186/s12893-020-00984-5

**Published:** 2020-12-02

**Authors:** Jing Zheng, Yuru Lan, Qiang Fan, Yunfei Ling, Yongjun Qian

**Affiliations:** 1 Anesthesia Operation Center of West China Hospital/West China School of Nursing, Sichuan University, Guoxuexiang 37th, Chengdu, 610041 Sichuan People’s Republic of China; 2 Department of Cardiovascular Surgery, West China Hospital, Sichuan University, Guoxuexiang 37th, Chengdu, 610041 Sichuan People’s Republic of China

**Keywords:** Anomalous aortic origin of a coronary artery, Neo-ostium creation, Lateral pulmonary translocation, Case report

## Abstract

**Background:**

Anomalous aortic origin of the coronary artery (AAOCA) is a rare congenital cardiac disease that can cause sudden cardiac death. This condition may be corrected with surgery. Among the different surgical techniques used to correct this malformation, the most common are unroofing and lateral pulmonary translocation.

**Case presentation:**

Herein, we present a multimodal imaging approach to identifying AAOCA in a 12-year-old male. We also successfully adopted a new operative method, neo-ostium creation combined with lateral pulmonary translocation to correct AAOCA. The detailed imaging and intraoperative data has not been reported in the literature.

**Conclusions:**

Although several surgical methods exist to reverse the complications of AAOCA, we offer an innovative surgical technique that is easier, faster, and effective.

## Background

Anomalous aortic origin of a coronary artery (AAOCA) is a rare congenital heart condition wherein a coronary artery originates from the wrong aortic sinus and follows an interarterial, intramural, or intraconal course [1]. While AAOCA is rare, it is the second leading cause of death in young athletes, The incidence rate is about 0.01%–0.2 [2]. All types of AAOCA are associated with the risk of coronary flow obstruction and myocardial ischemia, which may lead to sudden cardiac death [1]. Studies have shown that the right coronary artery is more involved in the incidence of AAOCA than the left coronary artery [3]. However, AAOCA involving the left coronary artery carries a higher risk [4].

The current treatment guidelines for AAOCA recommend activity restriction and, in case of an increased risk, surgical intervention [5]. In the presence of an intramural segment, the unroofing surgery is performed [6]. However, to our knowledge, a surgery that combines both neo-ostium creation and lateral pulmonary translocation in patients with AAOCA has not been reported. This report describes a rare case of a 12-year-old male with AAOCA who successfully underwent this innovative combined surgery (neo-ostium creation and lateral pulmonary translocation). Additionally, it details imaging and intraoperative information to provide an alternative approach to treating AAOCA.

## Case presentation

**Fig. 1:**
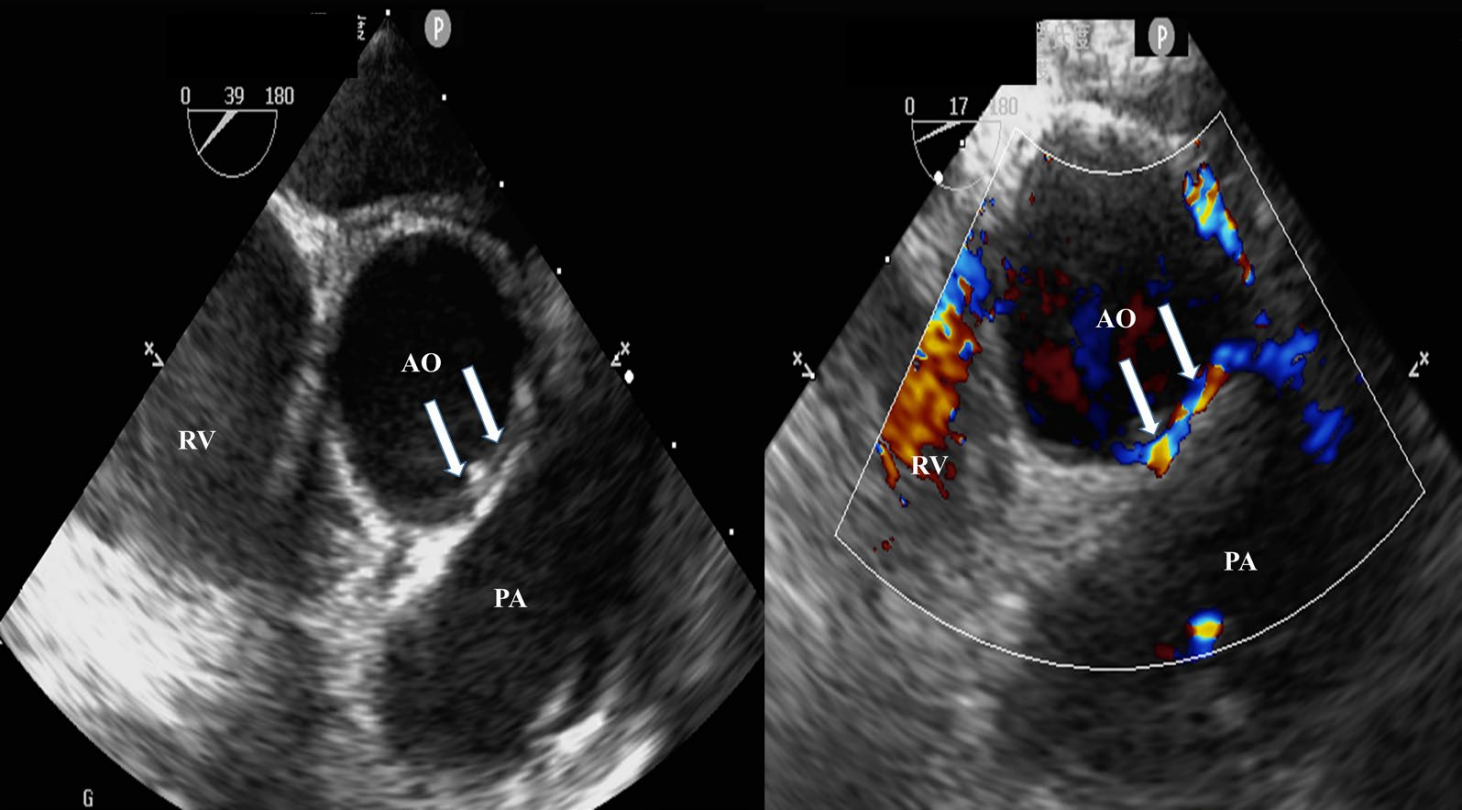
Transoesophageal echocardiography revealed the anomalous LCA and RCA arising more posteriorly and leftward from the right sinus of Valsalva and coursing intramural within the anterior aortic wall (arrows) between the Ao and PA. *LCA* left coronary artery, *RCA* right coronary artery, *Ao* aorta, *PA* pulmonary artery

**Fig. 2:**
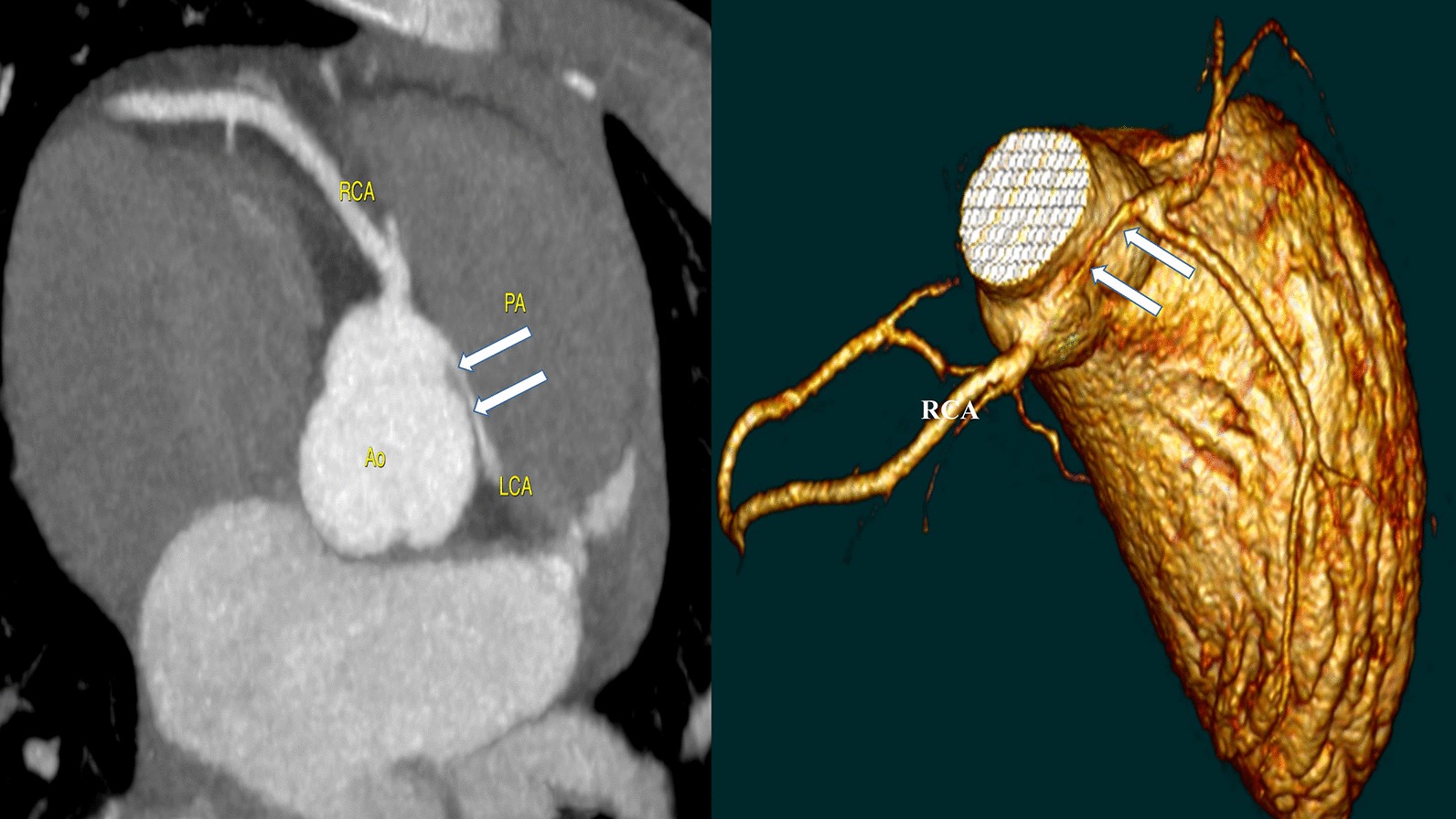
Computed tomography (CT) confirmed the diagnosis and showed the intramural course of the LCA with normal RCA, LAD and LCx. *LCA* left coronary artery, *RCA* right coronary artery, *LAD* left anterior descending artery, *LCx* left circumflex artery

A 12-year-old male was admitted to the hospital following sudden syncope after strenuous activity. The echocardiography showed the color in the left coronary artery. ECG revealed that the sinus rhythm was missing the ST segment and alterations in the T wave were indicative of ischemia. The lab analyses revealed that troponin, BNP, and fasting blood glucose levels were within normal levels. Transoesophageal echocardiography revealed that both the anomalous left coronary artery (LCA) and the normal right coronary artery (RCA) arose from a more posterior and leftward location on the right sinus of Valsalva and coursed intramurally along the anterior aortic wall (arrows) between the aorta (Ao) and the pulmonary artery (PA) (Fig. 1). Computed tomography (CT) confirmed the diagnosis and demonstrated the intramural course of the anomalous LCA with normal RCA, along with normal left anterior descending artery (LAD) and normal left circumflex artery (LCx) (Fig. 2).

Due to the AAOCA diagnosis, we next evaluated the patient for myocardial ischemia, disease progression, and surgical risk. To correct the malformation, we performed a novel surgery wherein a neo-ostium was created on the left coronary sinus while maintaining the abnormal opening. Additionally, we introduced a lateral translocation of the pulmonary artery to improve coronary flow.

**Fig. 3:**
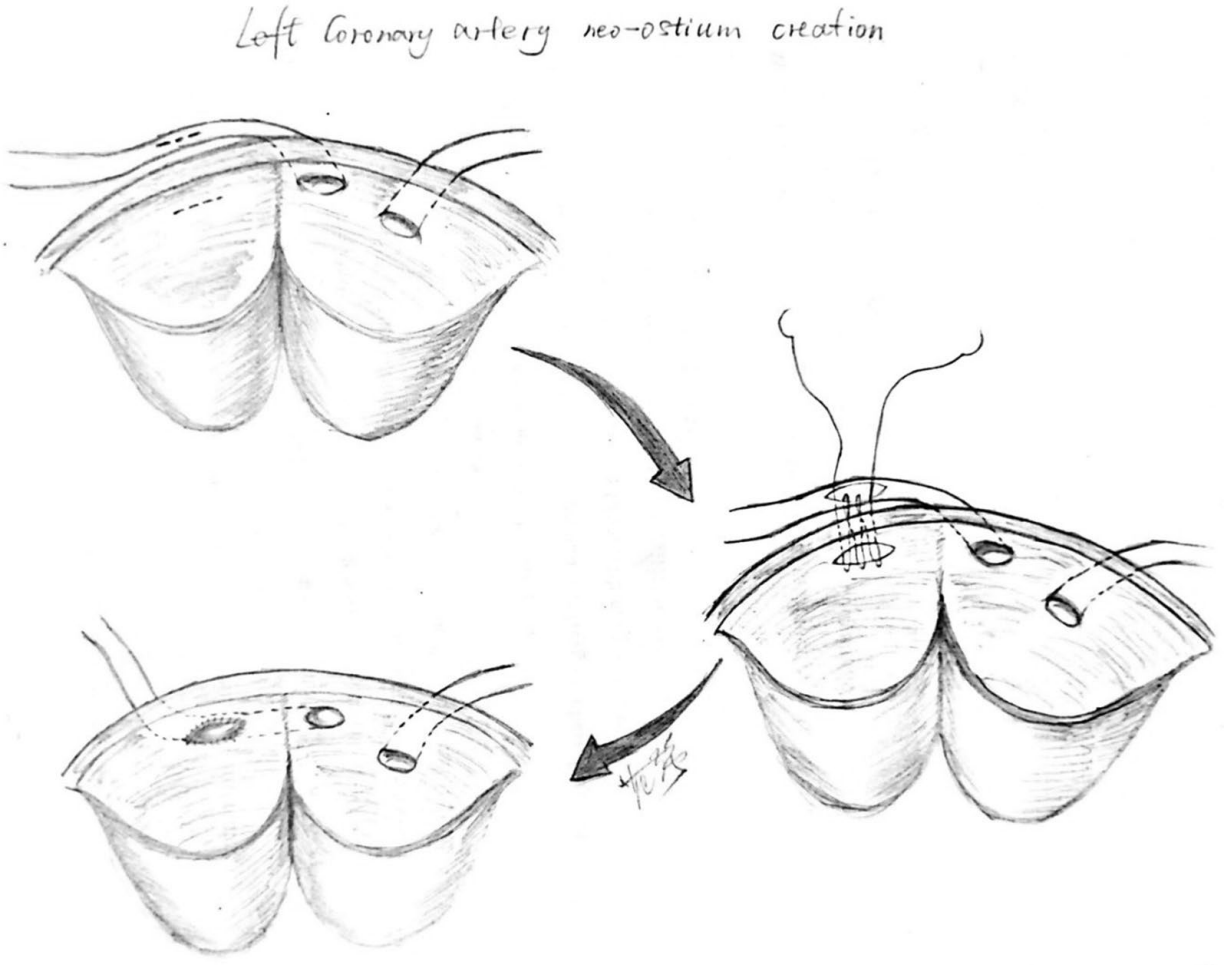
Illustration of the left coronary artery Neo-Ostium Creation procedure (graph by MD. Fan Qiang).Crosswise incisions were made in the distal of the narrow segment of left coronary artery and the corresponding position of the left coronary sinuses respectively and then anastomosed

**Fig. 4:**
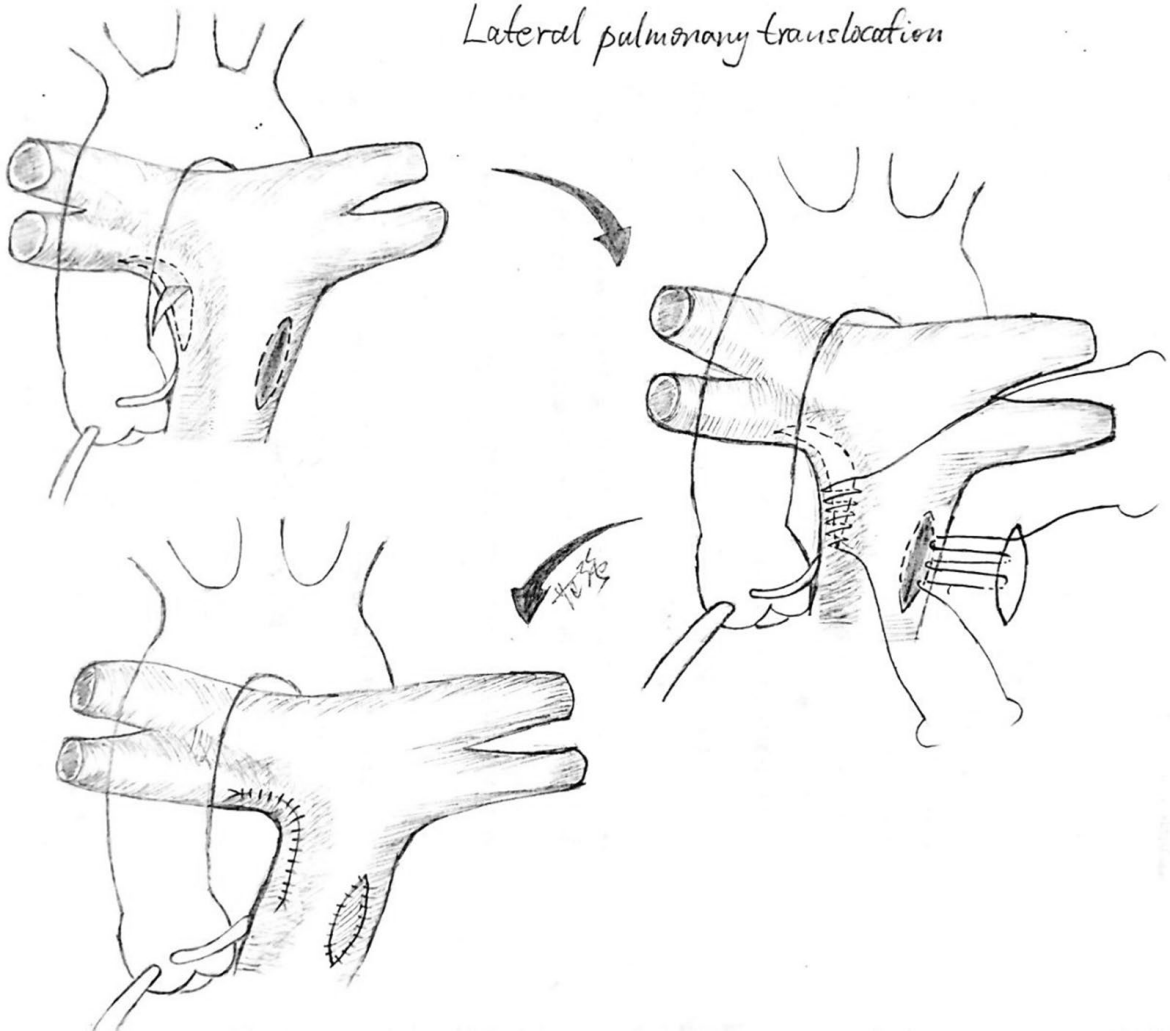
Illustration of the Lateral Pulmonary Translocation procedure (graph by MD. Fan Qiang). The pulmonary artery translocation was performed by introducing an approximately 1 cm long and 0.5 cm wide autologous blood vessel patch of the right pulmonary artery and inserting this patch in the lateral aspect of the main pulmonary artery

**Fig. 5:**
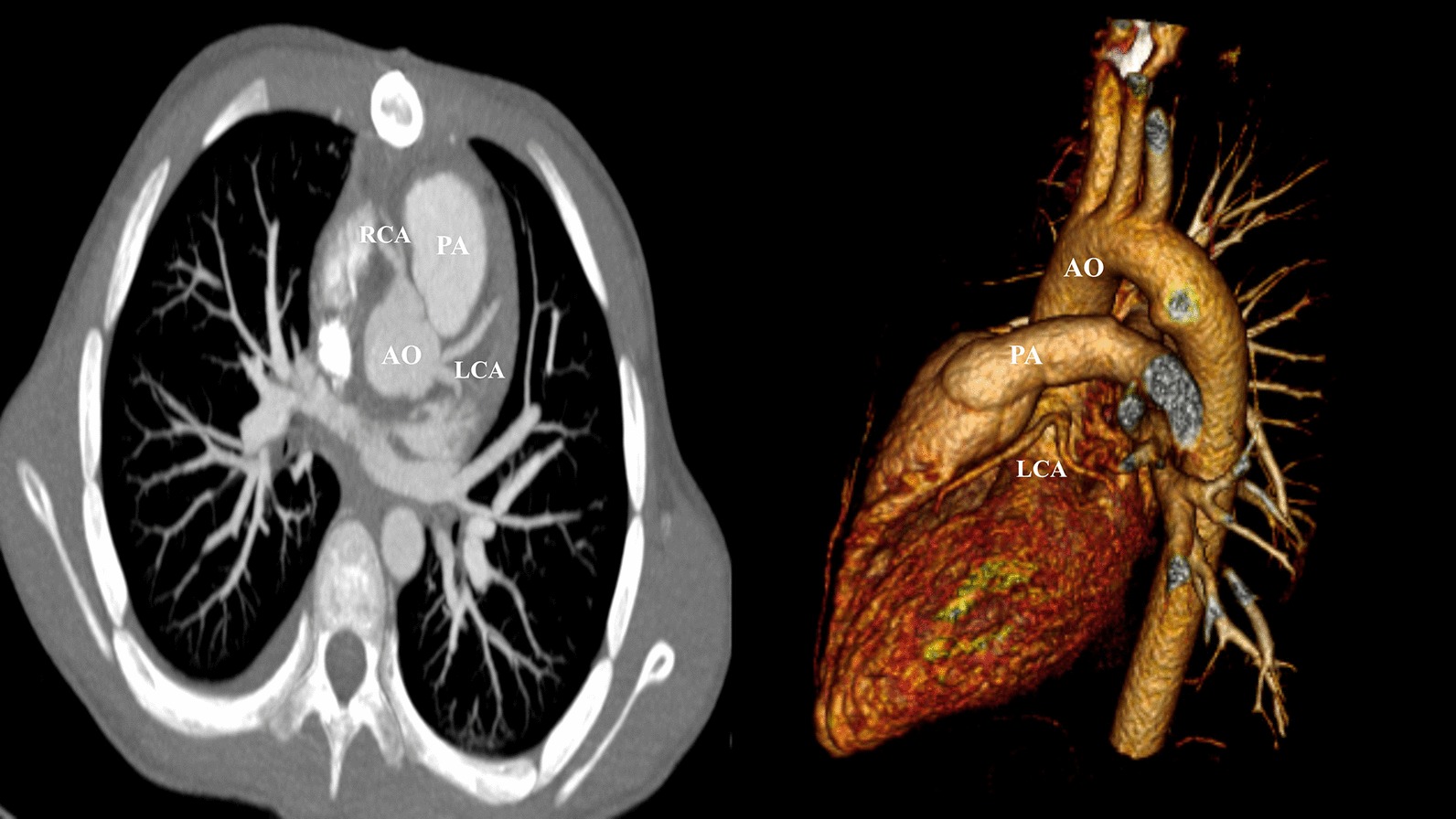
Postoperative CT revealed no stenosis of LCA and no interarterial compression

The surgery was performed with a median sternotomy. Cardiopulmonary bypass was initiated through aortic and bicaval cannulation. Antegrade cardioplegia was administrated to induce cardiac arrest. Crosswise incisions were made distal to the narrow segment of the left coronary artery and to the corresponding position of the left coronary sinuses respectively. Both locations were then anastomosed (Fig. 3). The pulmonary artery translocation was performed by introducing an approximately 1 cm long and 0.5 cm wide autologous blood vessel patch of the right pulmonary artery and inserting this patch in the lateral aspect of the main pulmonary artery (Fig. 4). After surgery, the patient was stable during the postoperative period and was discharged from the hospital uneventfully on the sixth day post operation. Postoperative CT revealed no stenosis of LCA and no interarterial compression (Fig. 5).

## Discussion and conclusions

AAOCA is a rare congenital cardiac disease wherein misplacement of a coronary artery can lead to blood flow obstruction and myocardial ischemia. Epidemiological data show that the prevalence of AAOCA is between 0.01 and 2% [7]. AAOCA is associated with sudden cardiac death [8], which was the 5th most common cause of sudden cardiac death. While the most common surgical procedure in treating AAOCA is unroofing of the intramural segments in the affected artery, the effectiveness and prime timing of the surgery have not yet been clearly defined [9].

In general, the decapitation of the coronary artery is carried out by incisively removing the common wall (the “roof”) of the artery, thus creating a larger new opening within the artery. Subsequently, clinical osteoplasty, transposition and reimplantation can be used as alternatives, particularly in the absence of intramural segments [8, 9].

PA translocation is an alternative option for treating patients with anomalous aortic origin of a coronary artery, a single coronary artery ostium, and no intramural component. This procedure can also be used to supplement an unroofing procedure when there is still a possibility of interarterial compression. In our case, instead of the conventional surgery where the coronary artery common wall is removed, we chose a new operational method, which introduces a new opening in the left coronary sinus while allowing the original abnormal opening to remain. Due to this variation, we also performed a lateral pulmonary artery translocation which was different from traditional PA translocation including LeCompte maneuver and lateral pulmonary artery translocation [10].

In summary, in clinical practice, patients with AAOCA usually undergo the method of unroofing to reduce the risk of sudden cardiac death, but other methods can also be adopted. A new, easier, and faster surgical technique, like the one proposed in this case report, may prove more helpful for patients with AAOCA.

## Data Availability

The datasets used in the case are available from the corresponding author upon reasonable request.

## Abbreviations

RA: Right atrium
LA: Left atrium
RV: Right ventricle
LV: Left ventricle
AO: Aorta
PA: Pulmonary artery
LCA: Left coronary artery
RCA: Right coronary artery
LAD: Left anterior descending artery
LCx: Left circumflex artery
AAOCA: Anomalous aortic origin of a coronary artery
